# Testing the Attentional Dwelling Hypothesis of Attentional Capture

**DOI:** 10.5334/joc.48

**Published:** 2018-10-03

**Authors:** Dominique Lamy, Maia Darnell, Adva Levi, Carmel Bublil

**Affiliations:** 1 Tel Aviv University, IL

**Keywords:** Attention, Visual search, Visual perception

## Abstract

Researchers are strongly divided as to whether abrupt onsets capture spatial attention in a purely stimulus-driven fashion or contingent on their search goals. Recently, Gaspelin, Ruthruff and Lien (2016) offered a resolution of this debate by showing that whether spatial capture by abrupt onsets is observed in a spatial cueing search task critically depends on search difficulty. To account for these findings, they proposed an “attentional dwelling” hypothesis, according to which, following capture by a cue, attention dwells at the cued location until the object subsequently appearing at that location is identified as the target or rejected as a distractor. A critical prediction of this account is that the more similar to the target the distractor at the cued location, the longer attention should dwell *at its location*. Yet, Gaspelin et al. (2016) did not test this prediction because they manipulated *overall search difficulty* rather than the difficulty of rejecting a specific distractor. The present study provides a critical test of the attentional dwelling hypothesis, by also varying target-distractor similarity within a trial rather than only between trials. Although we closely replicated these authors’ findings, the dwelling hypothesis passed the critical test in one of our two experiments. To accommodate the entire pattern of results observed here, we tentatively suggest a priority-accumulation framework, according to which cue validity effects do not necessarily index spatial shifts of attention, but instead, how much the cue speeds the resolution of the competition between the target and distractors in the search display.

One of the most debated issues in attention research concerns the factors that control attention. Although it makes intuitive sense to assume that attentional priority is jointly determined by how salient an object is and how relevant it is to our current goals, research in the past 30 years or so has painted a confusing picture (e.g., see Burnham, 2007; Lamy, Leber & Egeth, 2012; Rauschenberger, 2003; Theeuwes, 2010, for reviews), with two camps opposing each other. On the one hand, proponents of the salience-based view claim that some salient stimuli automatically summon attention, irrespective of the observer’s goals (e.g., Franconeri & Simons, 2003; Jonides & Yantis, 1988; Schreij, Owens & Theeuwes, 2008; Theeuwes, 2010; Theeuwes, Atchley & Kramer, 2000). On the other hand, proponents of the contingent-capture view suggest that only stimuli matching the observer’s current goals (or attentional set) attract attention (e.g., Ansorge, Horstmann & Scharlau, 2011; Bacon & Egeth, 1994; Carmel & Lamy, 2014; Eimer & Kiss, 2008, Folk, Remington & Johnston, 1992; Lamy, Leber & Egeth, 2004; Wu, Remington & Folk, 2014).

The status of abruptly onset objects (e.g., sudden flashes of light) as a special class of stimuli has been particularly central in this debate. Several authors have suggested that abrupt onsets have the unique ability to capture attention irrespective of the observer’s intentions (e.g., Jonides & Yantis, 1988). In contradiction to this view, other authors argue that abrupt onsets capture attention only if the observer’s attentional set includes searching for abruptly onset objects (e.g., Folk et al., 1992) or displays (e.g., Gibson & Kelsey, 1998) – although some of these authors concede that infrequent or unexpected onsets can capture attention in a strictly stimulus-driven fashion (e.g., Folk & Remington, 2015; Neo & Chua, 2006; but see Noesen, Lien & Ruthruff, 2014).

Recently, Gaspelin, Ruthruff and Lien (2016) made a significant step towards resolving the inconsistencies that exist in the extant literature with regard to the ability of abruptly onset objects to capture attention in a purely stimulus-driven fashion. Based on an extensive review, they noted that most studies using an easy search for color failed to observe capture, whereas most studies using a more difficult search for letters reported capture. This observation led them to suggest that onsets might always capture attention but the impact of such capture might critically depend on search difficulty.

To test this hypothesis, they conducted a series of spatial cuing experiments, while systematically manipulating search difficulty by varying target-distractor similarity. For instance, in Experiment 7, participants searched for a perfect circle among elliptical shapes. A black dot appeared on either the left or right inside of each shape in the display. Participants had to report the side of the dot in the target circle. On any given trial, all ellipses could be close, moderately far or far from perfect circularity, thereby yielding randomly mixed high-, medium- and low-similarity distractor trials, respectively. Prior to the search display, an abrupt onset was flashed randomly at one of the potential target locations. Attentional capture by the abrupt onset was measured by the validity effect, that is, better performance when the target appeared at the same location as the onset cue than at a different location. In line with the authors’ predictions, search times increased gradually with target-distractor similarity, (confirming that increasing target-distractor similarity increased search difficulty) and crucially, so did the validity effect.

To explain these findings, Gaspelin et al. (2016) proposed an “attentional dwelling” hypothesis. This account posits that following capture by a cue, attention remains at the cued location until the target display appears, even though the cue is spatially uninformative.^1^ It further postulates that attention dwells at the cued location until the object at that location is identified as the target or rejected as a distractor. This account thus explains why validity effects are larger in difficult search: RTs on different-location trials, which index attentional dwelling, are slower the more similar the distractors are to the target. Previous research has already suggested that the time taken to process and reject a distractor at an invalid-cue location strongly modulates the spatial cueing effect. For instance, Eriksen and Yeh (1985) showed that with imperfectly predictive abrupt-onset cues the RT cost on invalid-cue trials relative to an uninformative-cue condition was much larger when the target was surrounded by distractor letters (Exp. 1) than when it appeared alone in the display (Exp. 2). Prinzmetal, Ha and Khani (2010) reported similar results using utterly uninformative abrupt onset cues and comparing displays in which all placeholders at non-target locations were either empty (Exp. 3A) or filled with distractor letters (Exp. 3B). However, Gaspelin et al. (2016) provide two major novel contributions to this literature. First, they reviewed previous studies investigating stimulus-driven capture by abrupt onsets and showed that differences in target-distractor similarity can account for the diverging findings. Second, their design was better suited to testing the dwelling hypothesis: they manipulated target-distractor similarity gradually and within the same experiment rather than by contrasting filled vs. empty non-target locations between experiments.

Theeuwes and colleagues (e.g., Schreij et al., 2008; Theeuwes, 2010; Theeuwes et al., 2000) also suggested that how fast attention can be disengaged from the cued location determines whether attentional capture can be measured by the validity effect. However, Gaspelin et al.’s dwelling hypothesis differs from Theeuwes’ fast disengagement hypothesis in two notable respects. First, the former posits that attention dwells at the cued location and is disengaged from an invalidly cued location *after* target display onset, whereas the latter assumes that such disengagement starts already after the cue display and *before* target display onset. In addition, according to the former, disengagement speed is determined by the similarity between the *distractors* and target features, whereas according to the latter it is determined by the similarity between the *cue* and target features.

Accordingly, the two accounts essentially address different questions: the dwelling hypothesis’ aim is to explain why stimulus-driven capture by abrupt onsets is reported in some studies and not in others, whereas the fast-disengagement hypothesis’ aim is to explain why goal-driven attentional capture is more likely to be detected than stimulus-driven capture. Therefore, the two hypotheses are not necessarily incompatible. Disengagement from the cued location could start before target display onset and be faster when the cue does not match the target feature than when it does. In addition, if disengagement is incomplete during the cue-to-target interval, further dwelling at the cued location could depend on the similarity between the object at the cued location and the target feature. The objective of the proposed study was to investigate the dwelling hypothesis and testing it against the fast-disengagement hypothesis was therefore beyond its scope.

Gaspelin et al.’s (2016) findings and attentional dwelling hypothesis have important implications for the field of attentional capture research. They offer an elegant resolution of the long-standing controversy surrounding the status of abrupt onsets in attentional capture and contradict the strong version of the contingent-capture model by positing that a class of salient stimuli capture attention in a purely stimulus-driven fashion. They also have broader implications for our understanding of the mechanisms underlying spatial orienting. In particular, they suggest that the cost of reorienting attention from a distractor’s location to the target’s location mainly indexes the time required to decide whether or not the object at the locus of attention is the target, with the actual movement of attention from the distractor’s location to the target’s location incurring little or no cost at all (see also Eriksen & Yeh, 1985).

## Rationale for the present study

It should be noted that in all their experiments, Gaspelin et al. (2016) manipulated *overall search difficulty* (by varying target-distractor similarity either between blocks of trials or between trials) and so did earlier research (e.g., Eriksen & Yeh, 1985; Prinzmetal et al., 2010). This manipulation does not provide a direct test of the attentional dwelling hypothesis, which specifically predicts that the longer it takes to reject the distractor at the cued location, the longer attention dwells *at its location*, and the larger the observed validity effect should be. It is also noteworthy that when conditions of search difficulty were mixed *valid*-cue trials were substantially slower on difficult- than on easy-search trials (598 ms vs. 551 ms in Exp. 6 and 855 ms vs. 624 ms in Exp. 7). Thus, search difficulty inflated RTs in all conditions (including on valid-cue trials on which attentional dwelling at distractors’ locations was unlikely to occur) and the larger validity effects observed during difficult search may therefore be a by-product of higher overall RTs.^2^ Taken together, these observations suggest that Gaspelin et al.’s (2016) findings might merely reflect that attentional capture by onsets is more likely to be observed when search is difficult than when it is easy, with no specific role for the duration of attentional dwelling at the location of the cued distractor.

It is also noteworthy that the most striking modulation of the cue validity effect by target-distractor similarity in Gaspelin et al.’s (2016) study emerged in Experiment 7, with a validity effect of 28 ms vs. 144 ms in the low- vs. high-similarity conditions, respectively. However, unlike in the remaining experiments of the same study, in which such modulation was more modest, the target was a singleton in all conditions. Thus, participants may have used the strategy of searching for the singleton (Bacon & Egeth, 1994) and the validity effects reported in that experiment may reflect goal-directed capture by the abrupt onset.

The objective of the present study was to test the attentional dwelling account directly by varying distractor similarity also *within the search display* rather than only *between trials*. The first experiment was similar to Gaspelin et al.’s experiment (2016, Exp. 7) described earlier, with the following changes. (a) We used only two levels of target-distractor similarity (corresponding to the low-similarity and high-similarity ellipses used by Gaspelin et al., 2016) instead of three levels. (b) There were three search conditions: two conditions in which the distractors in the display all had the same shape, either similar or dissimilar to the target (all-difficult vs. all-easy search, respectively) as in Gaspelin et al. (2016) study, and an additional search condition, in which displays contained one similar (henceforth, difficult) distractor and two dissimilar (henceforth, easy) distractors (mixed-difficulty search condition). (c) All analyses were conducted on log transformed data in order to eliminate the impact of overall RT baseline differences on differences in the magnitude of the validity effect. In Experiment 2, the target was defined by its color. This experiment was similar to Gaspelin et al.’s (2016, Experiment 6), except that as in Experiment 1, a mixed-difficulty search condition was added to the all-easy and all-difficult search conditions.

We expected to replicate Gaspelin et al.’s (2016) finding and to observe larger cue-validity effects following the onset cue in the all-easy than in the all-difficult search condition. More crucially, the attentional dwelling hypothesis postulates that on trials in which a distractor appears at the cued location, the more similar to the target this distractor is on the target-defining dimension (here, shape in Experiment 1 and color in Experiment 2), the longer attention should dwell at its location. It therefore predicts that in the mixed-difficulty search condition, RTs should be slower when the location of the difficult distractor is cued than when the location of the easy distractor is cued.

Note that the above rationale assumes that following an involuntary attentional shift to an invalid-cue location, attention is reoriented directly towards the target rather than serially scanning the display until the target is found. Yet, it may not necessarily be the case. For instance, in the critical mixed-difficulty search condition, if attention is summoned by the cue to the location of an easy distractor, it may then be erroneously shifted to the location of the difficult distractor (because it resembles the target). Such detour of attention is less likely to occur when attention is summoned to the location of a difficult distractor, because in that case, the target is highly discriminable from the remaining distractors. According to this scenario, RTs may actually be slower on easy- relative to difficult cued-distractor trials (or just as slow if erroneous redirection of attention to the difficult distractor occurs only on part of the trials), because two shifts of attention may be required in the former condition and only one in the latter. As a result, while finding slower RTs on difficult- than on easy cued-distractor trials would support the dwelling hypothesis, alternative outcomes would not necessarily invalidate it.

Comparing the cue validity effect on difficult cued-distractor trials of the mixed-difficulty search condition vs. easy cued-distractor trials of the all-easy search condition allowed us to resolve this issue. In both conditions, all locations other than the cued location were occupied by easy distractors, such that the cost of attentional misdirection, if any, following capture, should be the same in the two conditions. In addition, the contribution of any general RT difference related to differences in overall display characteristics (e.g., distractor homogeneity vs. heterogeneity) and unrelated to dwelling should also accrue to valid-location trials and be therefore eliminated by its subtraction in the cue validity effect and the log transformation of the RT. Thus, the difference between these two conditions should specifically reflect differences in dwelling at the location of a difficult vs. easy distractor.

Finally, we tested an additional possible implication of the dwelling hypothesis in exploratory analyses. According to the dwelling account, attention dwells longer at the location of a distractor when this distractor is similar to the target than when it is dissimilar from it. One may expect that the longer attention dwells at a location, the more processing the object at that location it likely to receive. If so, more processing should accrue at the location of a cued distractor when this distractor is difficult than when it is easy. We tested this prediction by measuring the compatibility effect (e.g., Carmel & Lamy, 2014; Folk & Remington, 2006). In the present context, this effect refers to better performance when the response associated with the feature in the cued distractor (e.g., left or right dot in Experiment 1) is compatible with the response associated with the target (left or right dot) than when it is incompatible with it. We predicted that participants should be more likely to process the location of the dot inside the cued distractor when this distractor was difficult than when it was easy, and that as a result, compatibility effects should be larger in the former than in the latter condition.

## Experiment 1

### Method

#### Participants and sample size selection

We calculated the sample size required in order to observe a significant overall cue validity effect based on the effect size reported by Gaspelin et al. (2016, Experiment 7). We conducted this analysis with G*Power (Faul, Erdfelder, Buchner & Lang, 2013), using an alpha of 0.01 and power of 0.95. We found the minimum sample size required to be 6 participants. Nevertheless, in order to use a sample size similar to Gaspelin and al.’s, the experiment included 24 participants. Only participants who reported normal or corrected-to-normal visual acuity were allowed to take part in the experiment. Ethics approval was obtained before performing this and the following experiment.

#### Apparatus

The experiment took place in a dimly lit room. Stimuli were presented on a 23-in. LED screen, using 1,920 × 1,280 resolution graphics mode and 120-Hz refresh rate. Responses were collected via the computer keyboard. Viewing distance was set at approximately 60 cm from the monitor.

#### Stimuli

**Figure 1:**
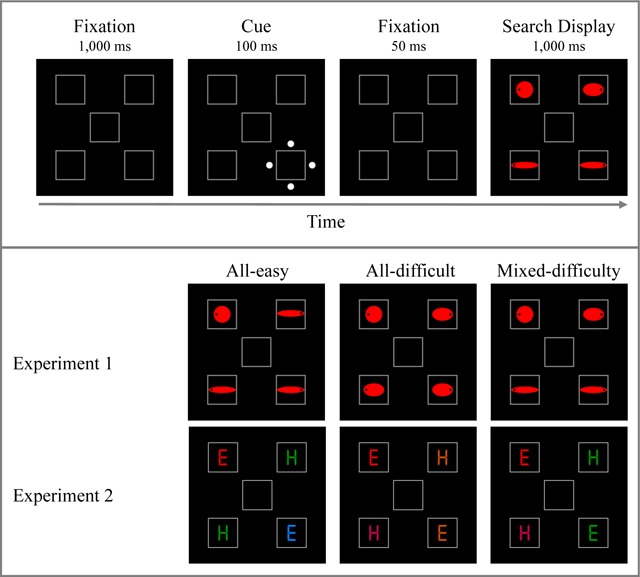
*Upper panel*: Sample sequence of events in Experiment 1. Participants searched for the perfect circle in the target display and reported the side of the black dot inside the target (left or right). This example corresponds to an invalid-cue trial in the mixed-difficulty search condition. In this example, the cued distractor is an easy distractor and the response associated with it (right) is incompatible with the response associated with the target (left). *Lower panel*: Sample displays in each search type condition (all-easy, all-difficult, mixed-difficulty) in Experiments 1 and 2. In Experiment 2, participants searched for the red target and reported whether it was an E or an H. The depicted example corresponds to an E target.

A sample sequence of events is presented in Figure 1. The fixation display consisted of five gray (138, 138, 138) square outline placeholders (2.4° × 2.4°), one centered at fixation and the remaining four equally spaced at the corners of an imaginary square subtending 10° in diameter (i.e., central-frame center to outer-frame center distance will be 5°). The onset-cue and target displays were similar to the fixation display, except for the following changes. In the onset-cue display, a cue consisting of four white dots (255, 255, 255; 0.5° in diameter) forming an imaginary diamond (3.3° × 3.3°) was added around one of the four outer placeholders. In the target display, a filled red shape (255, 0, 0) appeared in the centre of each of the outer placeholders: one circle (the target, 1.3° in diameter) and three horizontal ellipses (the distractors). “Difficult” distractors subtended 1.6° × 1° and “easy” distractors subtended 2.1° × 0.5°. On fixed-difficulty search trials, all distractors were either difficult (all-difficult search) or easy (all-easy search). On mixed-difficulty search trials, each display contained one difficult distractor and 2 easy distractors. A black dot (0.1° in diameter) appeared on the left or right side of each shape (0.1° from the outside), with each display containing exactly 2 left-dot and 2 right-dot shapes.

#### Procedure

Participants were instructed to search for the circle target and report on which of its sides (left or right) a black dot appeared. They were asked to respond as fast and as accurately as possible by pressing the key Z with their left hands or M with their right hands on the computer keyboard, if the dot appeared on the left or on the right, respectively. Each trial began with the fixation display for 1000 ms, followed by the cue display for 100 ms and then again by the fixation display for 50 ms. Then, the target display appeared until response. Following an incorrect response, participants heard an error beep (225 Hz) for 300 ms.

#### Design

The experiment consisted of 64 practice trials, followed by 8 blocks of 80 trials each.^3^ All-difficult (25%), all-easy- (25%) and mixed-difficulty (50%) search trials were randomly mixed within each block of trials. Conditions of onset-cue location, target location and location of the black dot in each shape (left or right) were equiprobable and randomly mixed within each block of trials.

### Results

Reaction times (RT) faster than 200 ms or slower than 2000 ms (.07% of the trials) were excluded from all RT and error rate analyses. No participant met the conditions for exclusion (either an average RT or an average accuracy rate differing from the group’s mean by more than 3 standard deviations). Error trials (5.9% of the trials) were excluded from RT analyses. All RT analyses were performed on log transformed RTs.

#### Pre-registered analyses

##### Replication of Gaspelin et al. (2016)

**Figure 2:**
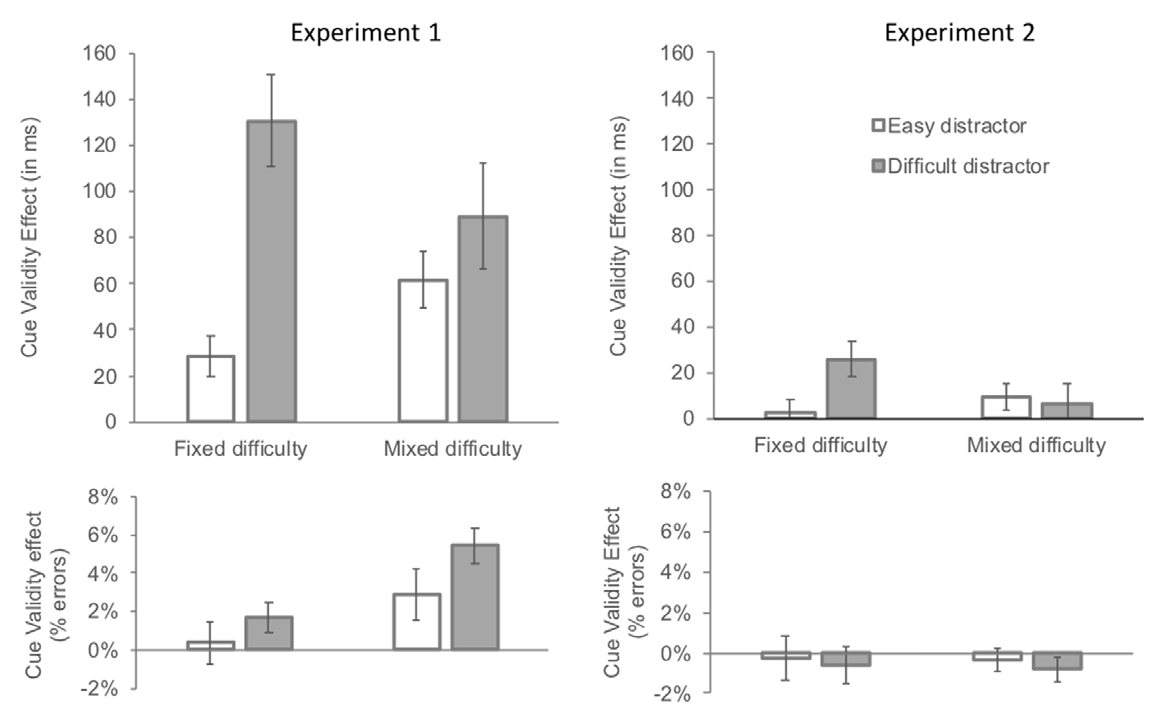
Mean cue validity effect (invalid-cue minus valid-cue) by search condition (fixed difficulty vs. mixed difficulty) and cued distractor difficulty (easy vs. difficult) in Experiment 1 (left panels) and in Experiment 2 (right panels). Note that the “fixed-difficulty easy” column corresponds to the all-easy search condition and the “fixed-difficulty difficult” column corresponds to the all-difficult search condition. *Upper panel*: Mean effect on reaction times (in milliseconds). *Lower panel*: Mean effect on error rates (in percentage).

We conducted an analysis of variance (ANOVA) with cue validity (valid vs. invalid) and search type (all-easy, all-difficult or mixed-difficulty) as independent variables. Mean validity effects on RTs and accuracy are presented in Figure 2. Raw RTs in the different conditions are presented in Table 1.

**Table 1:** Mean RTs (in milliseconds) and accuracy rates (in percentage) in Experiment 1 by conditions of cue validity, cued-distractor difficulty and search type. The numbers between square brackets represent the standard errors.

	All-easy		All-difficult		Mixed-easy		Mixed-difficult	

*Reaction times*								
Valid	608	[18]	787	[28]	696	[22]	696	[22]
Invalid	636	[22]	918	[30]	757	[22]	785	[23]
*Accuracy*								
Valid	96.1%	[1.1%]	95.1%	[1.2%]	96.1%	[1.0%]	96.1%	[1.4%]
Invalid	95.7%	[0.9%]	93.4%	[1.5%]	93.3%	[0.9%]	90.7%	[1.3%]

*Reaction times*. The main effects of cue validity and search type were highly significant, F(1, 23) = 33.7, p < .0001, *η^2^_p_* = .59, and F(1, 23) = 298.9, p < .0001, *η^2^_p_* = .93, respectively, and so was the interaction between these factors, F(1, 23) = 30.2, p < .0001, *η^2^_p_* = .57. Follow-up analyses revealed that the cue validity effect was larger on all-difficult than on all-easy search trials, F(1, 23) = 36.7, p < .0001, *η^2^_p_* = .61, and that the effect was significant in both conditions, 131 ms, F(1, 23) = 37.6, p < .0001, *η^2^_p_* = .62 and 28 ms, F(1, 23) = 12.6, p = .0017, *η^2^_p_* = .35 in the all-difficult and in the all-easy conditions, respectively. Thus, Gaspelin et al.’s (2016) main finding was closely replicated. The validity effect was of intermediate magnitude in the mixed-difficulty condition, 71 ms, F(1, 23) = 34.85, p < .0001 *η^2^_p_* = .60.

On valid-cue trials, the effect of search type was significant, F(2, 46) = 150.1, p < .0001, *η^2^_p_* = .87. Paired comparisons showed that, as in Gaspelin et al.’s (2016) study, valid-cue trials were faster in the all-easy than in the all-difficult search condition, 608 ms vs. 787 ms, F(1, 23) = 172.2, p < .0001, *η^2^_p_* = . 87. Reaction times on valid-cue trials in the mixed-difficulty search condition were intermediate, 696 ms: they were faster than on all-difficult search trials, F(1, 23) = 95.8, p < .0001, *η^2^_p_* = .88, and slower than on all-easy search trials, F(1, 23) = 14.37, p = .0009, *η^2^_p_* = .87.

*Accuracy*. The main effect of cue validity was significant, F(1, 23) = 6.5, p = .018, *η^2^_p_* = .22, and the main effect of search type was not, F(1, 23) = 2.2, p = 0.12, *η^2^_p_* = .09. The interaction between the two factors was significant, F(1, 23) = 4.7, p = .014, *η^2^_p_* = .17. Follow-up analyses revealed that the cue validity effect was larger in the mixed-difficulty search condition, 3.7%, than in either the all-difficult search condition, 1.7%, F(1, 23) = 4.5, p = .045, *η^2^_p_* = .16 or the all-easy search condition, .4%, F(1, 23) = 11.6, p = .0024, *η^2^_p_* = .16, with no difference between the latter two conditions, F < 1. The effect of search type on valid-cue trials was not significant, F < 1.

##### New tests of the dwelling hypothesis: dwelling at the location of an easy vs. difficult distractor

We first compared performance on mixed-difficulty search trials between the three cued location conditions (target, easy distractor, difficult distractor) in order to test the prediction that performance should be poorer when the cued distractor was difficult than when it was easy. Then, we compared the validity effect on all-easy vs. mixed-difficulty trials, with the latter including only difficult cued-distractor trials in the invalid-cue condition, such that apart from the cued distractors (easy vs. difficult), all remaining items in the two search conditions were identical: the target and two easy distractors.

*Reaction times*. The effect of cued location on mixed-difficulty search trials was significant, F(2, 46) = 36.8, p < .0001, *η^2^_p_* = .62. Planned comparisons revealed that the cue validity effect was significant both when the cued distractor was easy, 62 ms, F(1, 23) = 27.8, p < .0001, *η^2^_p_* = .55, and when it was difficult, 89 ms, F(1, 23) = 46.58, p < .0001, *η^2^_p_* = .67. Crucially, difficult cued distractor trials were slower than easy cued-distractor trials, 27 ms, F(1, 23) = 23.74, p < .0001, *η^2^_p_* = .51.

When comparing the validity effect with easy vs. difficult distractors in the all-easy vs. mixed-difficult search trials, respectively, the interaction between cue validity and search type was highly significant, F(1, 23) = 57.8, p < .0001, *η^2^_p_* = .72, indicating that the cue validity effect was larger when the location of a difficult distractor vs. of an easy distractor was cued, 30 ms vs. 89 ms, respectively, when the remaining distractors were identical in the two conditions.

*Accuracy*. The accuracy data closely mirrored the RT data. The effect of cued location on mixed-difficulty search trials was significant, F(2, 46) = 13.5, p < .0001, *η^2^_p_* = .37. Planned comparisons revealed that the cue validity was significant both when the cued distractor was easy, 2.9%, F(1, 23) = 8.8, p = .0069, *η^2^_p_* = .28 and when it was difficult, 5.4%, F(1, 23) = 17.3, p = .0004, *η^2^_p_* = .43. Crucially, difficult cued distractor trials were less accurate than easy cued-distractor trials, 2.5%, F(1, 23) = 10.21, p = .004, *η^2^_p_* = .31.

When comparing the validity effect with easy vs. difficult distractors on all-easy vs. mixed-difficult search trials, respectively, the interaction between cue validity and search type was highly significant, F(1, 23) = 16.5, p = .0005, *η^2^_p_* = .42, indicating that the cue validity effect was larger when the location of a difficult distractor vs. of an easy distractor was cued, 5.4% vs. .4%, respectively.

#### Exploratory analyses: compatibility effects

**Figure 3:**
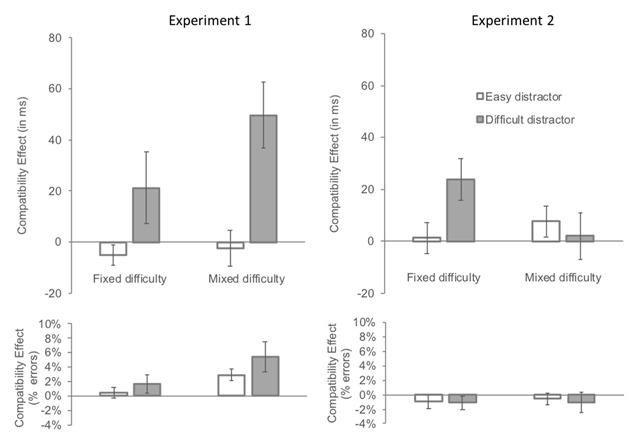
Mean compatibility effect (incompatible minus compatible) by search condition (fixed difficulty vs. mixed difficulty) and cued distractor difficulty (easy vs. difficult) in Experiment 1 (left panels) and in Experiment 2 (right panels). Note that the “fixed-difficulty easy” column corresponds to the all-easy search condition and the “fixed-difficulty difficult” column corresponds to the all-difficult search condition. *Upper panel*: Mean effect of reaction times (in milliseconds). *Lower panel*: Mean effect on error rates (in percentage).

We conducted an ANOVA with compatibility (compatible vs. incompatible), distractor difficulty (easy vs. difficult) and search type (fixed- vs. mixed-difficulty) as independent variables. Mean compatibility effects on RTs and accuracy are presented in Figure 3. Raw RTs in the different conditions are presented in Table 2.

**Table 2:** Mean RTs (in milliseconds) and accuracy rates (in percentage) on invalid-cue trials in Experiment 1 by conditions of cue-target compatibility, cued-distractor difficulty and search type. The numbers between square brackets represent the standard errors.

	All-easy		All-difficult		Mixed-easy		Mixed-difficulty	

*Reaction times*								
Compatible	640	[23]	904	[31]	759	[23]	753	[23]
Incompatible	635	[21]	925	[30]	757	[25]	803	[24]
*Accuracy*								
Compatible	96.1%	[1.1%]	95.1%	[1.2%]	96.1%	[1.0%]	96.1%	[1.4%]
Incompatible	95.7%	[0.9%]	93.4%	[1.5%]	93.3%	[0.9%]	90.7%	[1.3%]

*Reaction times*. The main effects of compatibility and cued distractor difficulty were significant, F(1, 23) = 5.0, p = .035, *η^2^_p_* = .18 and F(1, 23) = 373.5, p < .0001, *η^2^_p_* = .94, respectively, while the effect of search type was not, F < 1. All 2-way interactions were significant. The interaction between compatibility and distractor difficulty, F(1, 23) = 8.6, p = .0074, *η^2^_p_* = .27, indicated that the compatibility effect was larger when the cued distractor was difficult than when it was easy, 33 ms vs. 1 ms. The interaction between compatibility and search type, F(1, 23) = 6.6, p = .017, *η^2^_p_* = .22, indicated that the compatibility effect was larger when distractor difficulty was mixed than when it was fixed, 14 ms vs. 4 ms, respectively. Finally, the interaction between distractor difficulty and search type, F(1, 23) = 276.1, p < .0001, *η^2^_p_* = .92, indicated that the effect of distractor difficulty was larger in the fixed than in the mixed-difficulty condition, 281 ms vs. 27 ms, respectively. There was only a trend towards a three-way interaction, F(1, 23) = 3.4, p = .077, *η^2^_p_* = .13.

We nevertheless clarified this trend in order to compare the RT to the accuracy results (see below). We conducted separate analyses for each of the search type conditions. When distractor difficulty was fixed, the interaction between compatibility and distractor difficulty was not significant, F(1, 23) = 2.9, p = .1, *η^2^_p_* = .11, indicating that the compatibility effect was similar when the cued distractor was difficult, 21 ms, F(1, 23) = 1.5, p = .23, *η^2^_p_* = .06 and when it was easy, –5 ms, F(1, 23) = 2.1, p = .16, *η^2^_p_* = .08. When distractor difficulty was mixed, the interaction was significant, F(1, 23) = 10.4, p = .0038, *η^2^_p_* = .31, indicating that the compatibility effect was larger when the cued distractor was difficult, 50 ms, F(1, 23) = 13.5, p = .0013, *η^2^_p_* = .37 than when it was easy, 2 ms, F < 1.

*Accuracy*. The accuracy data closely mirrored the RT data. The main effects of compatibility and search type were significant, F(1, 23) = 8.5, p = .0077, *η^2^_p_* = .27 and F(1, 23) = 12.2, p = .002, *η^2^_p_* = .34, respectively, while the effect of cued distractor difficulty only approached significance, F(1, 23) = 3.8, p = .063, *η^2^_p_* = .14. Compatibility interacted with both distractor difficulty, F(1, 23) = 9.6, p = .0051, *η^2^_p_* = .29 and search type, F(1, 23) = 5.7, p = .026, *η^2^_p_* = .20, and these interactions were modulated by a significant three-way interaction, F(1, 23) = 20.1, p = .00017, *η^2^_p_* = .47.

To clarify this interaction, we conducted separate analyses for each search type condition. When distractor difficulty was fixed, the interaction between compatibility and distractor difficulty was not significant, F < 1, indicating that the compatibility effect was similar when the cued distractor was difficult, 0.4%, F < 1 and when it was easy, 0.9%, F(1, 23) = 1.3, p = .27, *η^2^_p_* = .05. When distractor difficulty was mixed, the interaction was significant, F(1, 23) = 17.4, p = .00037, *η^2^_p_* = .43, indicating that the compatibility effect was significantly larger when the cued distractor was difficult, 8.6%, F(1, 23) = 16.3, p = .00052, *η^2^_p_* = .41 than when it was easy, –2.1%, F(1, 23) = 6.5, p = .018, *η^2^_p_* = .22.

### Discussion

The results of Experiment 1 yielded several findings that support Gaspelin et al.’s (2016) dwelling hypothesis. First, the cue validity effect was much larger when all distractors were similar to the target than when they were dissimilar from it (all-difficult vs. all-easy distractor trials, respectively), thus closely replicating the finding reported by Gaspelin et al. Note that here unlike in Gaspelin et al. (2016, Exp. 7), the target was not a shape singleton on all trials (specifically, in the mixed-difficulty search condition). As all the search conditions were randomly mixed, singleton-detection mode was therefore an ineffective search strategy in our experiment and the validity effect observed here in the all-easy and in the all-difficult search conditions unambiguously indexed purely stimulus-driven attention capture.

Second, we confirmed the pivotal prediction of the dwelling hypothesis: when search displays contained both easy and difficult distractors (mixed-difficulty search trials), performance at responding to the target was poorer when the cued distractor was difficult than when it was easy. Third, exploratory analyses showed that processing at the location of the cued distractor, which was indexed by response compatibility effects, was more extensive when this distractor was difficult (and attention presumably dwelled longer at its location) than when it was easy.

Two aspects of these results are worth noting. One is that the validity effect for easy distractors was much larger on mixed-difficulty than on all-easy search trials, 62 ms vs. 29 ms, respectively. Conversely, the validity effect for difficult distractors was much smaller on mixed-difficulty than on all-difficult search trials, 89 ms vs. 130 ms, respectively. To account for these findings within the dwelling hypothesis framework, one may assume that after the onset cue captured attention, attention was more likely to be reoriented to the location of a difficult distractor than to the location of an easy distractor before it landed on the target location. Since there were more difficult distractors to redirect attention to on mixed-difficulty than on all-easy trials condition when an easy distractor was cued, and conversely in the all-difficult than on mixed-difficulty trials when a difficult distractor was cued, the validity effects were larger in the respective former conditions.

The second aspect is that the larger compatibility effect when the cued distractor was difficult than when it was easy was observed only during mixed-difficulty search. Again, this finding can be accounted for by relying on the assumption that attention was sometimes redirected to the location of a difficult distractor following attentional capture by the onset cue. Shifting attention to more than one difficult distractor before selecting the target may have diluted the compatibility effect in the all-difficult condition: for instance, attention could be captured at the location of a compatible-response distractor and then shifted to the location of an incompatible-response distractor. By contrast, such dilution was unlikely to occur following capture at the location of a difficult distractor on mixed-difficulty search trials, because there was no additional difficult distractor to shift attention to. This account yields the additional prediction that on mixed-distractor search trials, we should observe a compatibility effect from the difficult distractor even when it was not cued, since this account assumes that attention was often redirected to the difficult distractor after the location of an easy distractor was cued. A post-hoc analysis confirmed this prediction: RTs were faster when the response associated with the non-cued difficult distractor was compatible with the response associated with the target than when it was incompatible, 24 ms, F(1, 23) = 10.0, p = .0043, *η^2^_p_* = .30, with a similar effect on accuracy, 6.1%, F(1, 23) = 21.8, p = .0001 *η^2^_p_* = .49.

Note that the account invoked in both instances implies that on mixed-difficulty trials, attention was mis-oriented twice before reaching the target on easy cued-distractor trials (to an easy distractor and then to the difficult distractor) and only once on difficult cued-distractor trials (to the difficult distractor). Yet, the cue validity effect was significantly smaller in the former than in the latter condition (62 ms vs. 89 ms, respectively). Thus, the attentional dwelling hypothesis does not readily account for the full pattern of the results observed in Experiment 1.

## Experiment 2

The primary objective of Experiment 2 was to replicate the findings of Experiment 1 when the target was defined on the color dimension (which is more typical of previous attentional capture experiments) rather than on the shape dimension. The experiment was similar to Experiment 1 except for the following differences. Participants searched for a red-letter target among distractor colored letters. Unlike in Experiment 1, in the fixed-difficulty conditions, distractor colors were not uniform. Difficult distractors were either pink or orange. Easy distractors were either blue or green.

Note that the stimuli and procedure were closely modelled after those of Gaspelin et al. (2016, Exp. 6) in the all-easy and all-difficult conditions, which allowed us to ensure that we replicated their findings before testing our novel predictions. We thus expected to observe larger attentional capture by the onset cue in the all-easy than in the all-difficult search condition. Of main interest was whether we could replicate the findings of Experiment 1 that provided direct support for the dwelling hypothesis, namely, poorer performance on difficult than on easy cued-distractor trials in the mixed-difficulty search condition. We also examined whether the findings from the exploratory analyses conducted on the data of Experiment 1 could be replicated, namely, the larger compatibility effects when the cued distractor was difficult than when it was easy.

### Methods

The sample size, apparatus, stimuli, procedure and design were similar to those of Experiment 1 except for the following differences. In the target display, instead of an ellipse, a letter (either E or H) drawn in a digital-clock font and subtending 1.9° × 1.9° of visual angle appeared within each placeholder. Each display contained one red letter, the target. In the all-difficult search condition, one distractor was pink and the other two were orange, or vice-versa. In the all-easy search condition, one distractor was blue and the other two were green, or vice-versa. In the mixed-difficulty search condition, one distractor was either orange or pink (difficult distractor) and the other two were either both green or both blue (easy distractors). Each display contained exactly two Es and two Hs. Participants were required to determine whether the red letter was E or H.

### Results

Reaction times (RT) faster than 200 ms or slower than 2000 ms (.1% of the trials) were excluded from RT and error rate analyses. The data from one participant were excluded from all analyses because her accuracy rate was below the group’s mean by more than 3 standard deviations (M = 77% vs. M = 94.8%, SD = 3.1%). All RT analyses performed on log transformed RTs for correct-response trials.

#### Pre-registered analyses

##### Replication of Gaspelin et al. (2016)

We first conducted an ANOVA with cue validity (valid vs. invalid) and search type (all-easy, all-difficult or mixed-difficulty) as independent variables. Mean validity effects on RTs and accuracy are presented in Figure 2. Raw RTs in the different conditions are presented in Table 3.

**Table 3:** Mean RTs (in milliseconds) and accuracy rates (in percentage) in Experiment 2 by conditions of cue validity, cued-distractor difficulty and search type. The numbers between square brackets represent the standard errors.

	All-easy		All-difficult		Mixed-easy		Mixed-difficult	

*Reaction times*								
Valid	566	[12]	622	[19]	590	[16]	590	[16]
Invalid	568	[13]	648	[19]	599	[16]	596	[15]
*Accuracy*								
Valid	95.6%	[0.9%]	93.9%	[1.5%]	94.2%	[0.9%]	94.2%	[0.9%]
Invalid	95.8%	[0.6%]	94.6%	[0.9%]	94.6%	[0.8%]	95.0%	[0.7%]

*Reaction times*. The main effect of search type was significant, F(1, 22) = 43.5, p < .0001, *η^2^_p_* = .66, whereas the effect of cue validity was not, F(1, 22) = 1.5, p = .23, *η^2^_p_* = .06. The interaction between the two factors was significant, F(1, 22) = 3.2, p = .05, *η^2^_p_* = .13. Follow-up analyses revealed that the cue validity effect was larger on all-difficult than on all-easy search trials, F(1, 22) = 5.8, p = .025, *η^2^_p_* = .21, and approached significance on all-difficult search trials, 26 ms, F(1, 22) = 4.0, p = .056 *η^2^_p_* = .15^4^ but not on all-easy search trials, 3 ms, F < 1, respectively, thus replicating Gaspelin et al.’s (2016) main finding. The validity effect did not differ between the all-easy and mixed-difficulty conditions, 3 ms vs. 8 ms, F(1, 22) = 1.1, p = .31 *η^2^_p_* = .04.

On valid-cue trials, the effect of search type was significant, F(2, 44) = 23.2, p < .0001, *η^2^_p_* = .51. Paired comparisons showed that, as in Gaspelin et al.’s (2016) study, valid-cue trials were faster on all-easy than on all-difficult search trials, 566 vs. 622 ms, F(1, 22) = 15.7, p = .0007, *η^2^_p_* = .56. Valid-cue trials on mixed-difficulty search trials were intermediate, 590 ms: they were faster than on all-difficult search trials, F(1, 22) = 23.2, p < .0001, *η^2^_p_* = .46 and slower than on all-easy search trials, F(1, 22) = 15.7, p = .0007, *η^2^_p_* = .42.

*Accuracy*. Only the main effect of search type approached significance, F(1, 22) = 3.0, p = 0.059, *η^2^_p_* = .12, all other Fs < 1. The effect of search type on valid-cue trials was not significant, F(1, 22) = 1.8, p = .17, *η^2^_p_* = .08.

##### New tests of the dwelling hypothesis: dwelling at the location of an easy vs. difficult distractor

We compared performance on mixed-difficulty search trials between the three cued location conditions (target, easy distractor, difficult distractor).

*Reaction times*. The effect of cued location was not significant, F < 1, indicating that RTs did not differ when the target, easy distractor or difficult distractor were cued in the mixed-difficulty condition, 590 ms, 599 ms and 596 ms, respectively.

*Accuracy*. The effect of cued location was not significant, F = 1.

We then compared the validity effect on all-easy vs. mixed-difficulty search trials with the latter including only difficult cued-distractor trials in the invalid-cue condition. Thus, apart from the cued distractors (easy vs. difficult), all remaining items in the two search conditions were identical: the target and two easy distractors.

*Reaction times*. The interaction between cue validity and search type was not significant, F(1, 22) = 2.4, p = .13, *η^2^_p_* = .10, indicating that the cue validity effect did not differ whether the cued distractor was easy or difficult, 3 ms vs. 10 ms, respectively, when the remaining distractors were identical in the two conditions.

*Accuracy*. The interaction between cue validity and search type was not significant, F < 1.

#### Exploratory analyses: compatibility effects

We conducted an ANOVA with compatibility (compatible vs. incompatible), distractor difficulty (easy vs. difficult) and search type (fixed-difficulty vs. mixed-difficulty) as independent variables. Mean compatibility effects on RTs and accuracy are presented in Figure 3. Raw RTs in the different conditions are presented in Table 4.

**Table 4:** Mean RTs (in milliseconds) and accuracy rates (in percentage) on invalid-cue trials in Experiment 2 by conditions of cue-target compatibility, cued-distractor difficulty and search type. The numbers between square brackets represent the standard errors.

	All-easy		All-difficult		Mixed-easy		Mixed-difficult	

*Reaction times*								
Compatible	567	[14]	632	[20]	594	[16]	595	[15]
Incompatible	569	[14]	656	[20]	602	[16]	597	[15]
*Accuracy*								
Compatible	96.3%	[1.0%]	93.7%	[1.5%]	91.8%	[1.4%]	96.4%	[0.8%]
Incompatible	95.4%	[1.0%]	93.3%	[1.7%]	94.0%	[0.9%]	87.8%	[2.0%]

*Reaction times*. All main effects were significant, F(1, 22) = 6.5, p = .018, *η^2^_p_* = .23, for compatibility, F(1, 22) = 51.4, p < .0001, *η^2^_p_* = .70, for cued distractor difficulty, and F(1, 22) = 5.1, p = .034, *η^2^_p_* = .19, for search type. The two-way interactions between compatibility and distractor difficulty and between distractor difficulty and search type were significant, F(1, 22) = 5.2, p = .032, *η^2^_p_* = .19 and F(1, 22) = 44.9, p < .0001, *η^2^_p_* = .67, respectively, and were modulated by a significant three-way interaction, F(1, 22) = 5.8, p = .025, *η^2^_p_* = .21.

To clarify this interaction, we conducted separate analyses for each search type condition. When distractor difficulty was fixed, the compatibility effect was significantly larger when the cued distractor was difficult than when it was easy, F(1, 22) = 18.9, p = .0003, *η^2^_p_* = .46. Follow-up comparisons showed that the compatibility effect was significant when the difficult distractor was cued, 24 ms, F(1, 22) = 16.7, p = .0005, *η^2^_p_* = .43, but not when an easy distractor was cued, 1 ms, F < 1. When distractor difficulty was mixed, the compatibility effect did not differ whether the cued distractor was easy or difficult, F < 1 and was not significant is either condition, 8 ms, F < 1 and 2 ms, F(1, 22) = 1.8, p = .19, *η^2^_p_* = .08, respectively.

*Accuracy*. Only the main effect of compatibility was significant, F(1, 22) = 10.2, p = .004, *η^2^_p_* = .32, all other ps > .15.

### Discussion

In Experiment 2, we again replicated Gaspelin et al.’s (2016) main finding: the cue validity effect was larger when all distractors were similar to the target than when they were dissimilar from it (all-difficult vs. all-easy distractor trials, respectively). Crucially, however, on mixed-difficulty search trials, performance was similar irrespective of whether an easy or a difficult distractor was cued. Thus, the critical prediction of the dwelling hypothesis was not supported in the present experiment.

Compatibility effects (i.e., better performance when the responses associated with the cued distractor and target were compatible than when they were incompatible) were consistent with the pattern of results observed on cue validity effects. They were larger when the cued distractor was difficult than when it was easy, but only in the condition in which distractor difficulty modulated the cue validity effect, that is, in the fixed- and not in the mixed-difficulty condition. Note that this pattern is the opposite of what we observed in Experiment 1, where increasing distractor difficulty enhanced the compatibility effect when conditions of distractor difficulty were mixed and not when they were fixed. To account for the findings of Experiment 1, we had suggested that the compatibility effect was likely to be diluted when shifts between more than one difficult distractor were possible (in all-difficult search) than when they were not (in mixed-difficulty search). One could claim that such dilution on all-difficult search trials did not occur in Experiment 2 because search was much easier (with a mean overall RT of 600 ms vs. 755 ms, respectively) and attention may have been shifted directly to the target after it dwelled at the location of just one difficult distractor on all-difficult search trials. However, this account cannot explain why neither cue validity nor compatibility effects were observed for difficult distractors on mixed-difficulty trials.

## General discussion

The objective of the present study was to provide a direct test of the attentional dwelling account of attentional capture. Specifically, we argued that Gaspelin’s et al. (2016) manipulation of overall search difficulty did not allow them to test the core tenet of that hypothesis, according to which the magnitude of the cue validity is determined specifically by how long it takes to reject the distractor *at the cued location*. To test this prediction, we did not only vary distractor difficulty between search displays but also within search displays. To do that, we added a mixed-difficulty search condition, in which the search displays contained one difficult distractor and two easy distractors. We could thus examine whether, as predicted by the attentional dwelling hypothesis, RTs were slower when the location of the difficult distractor was cued than when the location of the easy distractor was cued.

### A re-evaluation of the attentional dwelling hypothesis

On the one hand, we closely replicated all Gaspelin et al.’s (2016) findings. In both a shape search (Exp. 1, which paralleled their Exp. 7) and a faster color search (Exp. 2 here, which paralleled their Exp. 6), the cue validity effect was larger when all distractors were similar to the target (all-difficult search trials) than when they were dissimilar from it (all-easy search trials). On the other hand, however, when conditions of distractor difficulty were mixed within the same displays, it took longer to reject the distractor at the cued location when it was difficult than when it was easy only in Experiment 1 and not in Experiment 2. Thus, the critical prediction of the attentional dwelling hypothesis was not fully supported in the present study.

It is noteworthy that the dwelling hypothesis does not offer a straightforward account for additional findings from the present study. First, it does not explain the largest effect we observed, namely, the dramatic slowing of performance on valid-cue trials as search difficulty increased – a finding that Gaspelin et al. (2016) also observed, as noted in the introduction, but did not discuss. The dwelling hypothesis stipulates that attention is captured to the location of the onset cue and remains at that location until after the target display has appeared. It further posits that response speed is determined by how long it takes to decide whether the object at the cued location is the target or a non-target. Within this framework, why should this decision take so much longer (180 ms in Exp. 1 and 57 ms in Exp. 2) when the target is cued and no further shift of attention should therefore be required, if the target is surrounded by difficult distractors than if it is surrounded by easy distractors?

Second, it does not explain why RTs were faster on difficult cued-distractor trials and slower on easy cued-distractor trials in the mixed-difficulty relative to the all-difficult search condition. If distractor difficulty determines dwelling time, no such difference should be expected between the search conditions.

Finally, the dwelling hypothesis does not explain the full pattern of results that arose from exploratory analyses of the compatibility effect, in particular, why this pattern was opposite in Experiment 1 vs. 2 and why a compatibility effect was associated with the difficult distractor on trials in which an easy distractor was cued in the mixed-difficulty search condition of Experiment 1.

The conclusion that arises from the foregoing discussion is that there are powerful context effects during spatial cuing search tasks that the attentional dwelling hypothesis does not readily accommodate.

### Do cue validity effects reflect spatial shifts of attention?

Together with most accounts of attentional capture (e.g., Carmel & Lamy, 2015; Folk et al., 1992; Schreij, Theeuwes & Olivers, 2010; Zivony & Lamy, in press), the attentional dwelling hypothesis assumes that cue validity effects index shifts of spatial attention. Put simply, the finding that performance is faster when the target appears at the location previously occupied by a cue is taken as evidence that attention was shifted to the location of this cue. The cue validity effect is thought to index the cost of disengaging attention from the cue, of rejecting the object at the cued location as a non-target and of shifting attention from the cued location to the target location, with different authors emphasizing each of these cost components to different extents. We suggest that in order to accommodate the present and past findings, it may be useful to reconsider this interpretation.

The alternative interpretation that we tentatively put forward can be articulated around three main hypotheses, that draw from Desimone and Duncan’s (1995) biased competition framework. (1) Several factors determine the attentional priority level of a given object in a search display, in particular, the extent to which this object itself and objects that recently appeared at its location (e.g., a cue), are similar to the searched-for target and how physically salient they are, as well as noise. (2) When the target’s attentional priority level is larger than that of any distractor in the search display, the target is the first object that receives attention. Nevertheless, how long it takes for the competition to be resolved varies as a function of the size of this attentional priority benefit. An important implication of this is that finding a cue validity effect does not necessarily entail that attention was shifted to the location of the cue. Instead, it may indicate that the extra activation that accrues to the target when its location rather than a distractor’s location is cued, speeds the resolution of the competition that leads to target selection. (3) When the target’s attentional priority level is not the largest, one or several distractors may successively become the focus of attention before the target is selected. In that case, the validity effect reflects several additional processes including the time it takes to reject the distractor(s) and shift attention to the target.

Note that the suggested framework can readily account for the highly replicated finding that target-matching cues capture attention both in easy and in difficult search (e.g., Folk et al., 1992; Zivony & Lamy in press). One need only make the reasonable assumption that the extra activation accruing to the object that appears at the location of a target-matching cue is usually so large that this object will win the competition in most cases.

This priority-accumulation framework departs from the attentional dwelling account in three fundamental aspects. First, it implies that in spatial cuing paradigms, it is not the case that a cue either captures attention or does not: instead, a cue increases the probability that attention will be allocated to the cued location, to different extents.^5^ Second, the priority-accumulation framework suggests that distractor difficulty modulates the *probability* that an abrupt onset will capture attention, whereas according to the dwelling account, distractor difficulty only affects *dwelling time*, after attention was captured. Finally, the suggested framework does not require the assumption that shifts of attention may take no time at all, as implied by the attentional dwelling account’s interpretation of null cue validity effects following capture by onsets in easy search (e.g., Gaspelin et al., 2016, Exp. 1).

### Reinterpretation of the current findings

The suggested interpretation of cue validity effects can accommodate all the findings of the present study. It accounts for the larger validity effects in the all-difficult than in the all-easy search condition because the stronger the competition between the target and distractors, the stronger impact the cue is likely to have on the outcome of this competition. It also accounts for the poorer performance on *valid-cue* trials as a function of overall distractor-target similarity in both experiments, because responses to the target are slower the more competition it suffers from surrounding distractors.

Finally, it accounts for the pattern of results in the mixed-difficulty condition. According to the suggested framework, the cue validity effect in this condition indexes the cost of shifting attention to the difficult distractor irrespective of whether the cued distractor is easy or difficult. Shifting attention to the difficult distractor is maximally likely to occur when the difficult distractor is cued, somewhat less likely when the easy distractor is cued and least likely when the target is cued. This explains the RTs in the three conditions in Experiment 1 (696 ms, 757 ms and 785 ms, respectively) as well as the finding that the difficult distractor produced a compatibility effect irrespective of whether it was cued or an easy distractor was cued. However, when the target’s priority advantage is large enough, the difficult distractor prevails in none of these conditions, hence the similar RTs in the three conditions observed in mixed-difficulty search condition in Experiment 2.

### Contradiction with the findings reported by Zivony and Lamy (in press)

It is noteworthy that the compatibility effects observed in the present study stand in contradiction with findings recently reported by Zivony and Lamy (in press). These authors conducted three experiments using cues and displays highly similar to those of the all-difficult search condition in the present Experiment 2. They found cue validity effects following onset cues both when these cues matched the attentional set (relevant-color cues) and when they did not (irrelevant-color cues). However, compatibility effects occurred only following the former cues, that is, they were absent following irrelevant-color onset cues (similar to those used in the present study).

Based on the assumption that cue validity effects index spatial shifts of attention to the cued location and compatibility effects index increased processing of the information at the cued location (i.e., attentional engagement), Zivony and Lamy (in press) concluded that attentional engagement is contingent on search goals and that attentional shifts can be shallow, that is, not followed by attentional engagement (e.g., after stimulus-driven capture). The present findings challenge these conclusions because we found instances of compatibility effects following irrelevant-color onset cues, namely, in the mixed-difficulty search condition of Experiment 1 and in the all-difficult search condition of Experiment 2. To resolve this contradiction, one may suggest that target-distractor similarity and irrelevant-color onset cue strength were such in Zivony and Lamy’s (in press) experiments, that the target was the first attended object in the search display (hence the absence of compatibility effects), but the extra-activation provided by the cue nevertheless speeded target selection (hence, the cue validity effects). However, further research is clearly required in order to test these speculations.

## Conclusions

The results from the present study reinforce Gaspelin et al.’s claim (2016) that search difficulty modulates cue validity effects in spatial cuing tasks. However, they do not support the attentional dwelling hypothesis, according to which abrupt onsets routinely capture attention and the similarity of the object at the cued location determines how long attention dwells at its location, with cue validity effects indexing such dwelling time. To account for the entire pattern of results observed here, we offered a tentative reinterpretation of cue validity effects, according to which these do not necessarily index spatial shifts of attention. Although the proposed framework can account for the current and past findings as well as generate interesting novel predictions, it is clearly speculative at this point and requires further validation.

## Data Availability

The data sets discussed in this paper can be accessed at https://doi.org/10.6084/m9.figshare.7285289.v1

## Footnotes

Findings from our lab are consistent with this claim. We found the effect of attentional capture by a spatially uninformative relevant-color cue to remain constant across stimulus onset asynchronies (SOAs) ranging from 50–60 ms to 250 ms (Carmel & Lamy, 2014; Lamy et al., 2004) and up to 600 ms (Gabbay, Asaly & Lamy, 2017), an effect we labelled “attentional inertia”. It is noteworthy, however, that our findings pertained to goal-directed attention since the cue shared the target color.

Gaspelin et al. (2016, p. 1117) reported indirect evidence against this alternative account. They showed that (a) the effect size of the validity effect increased as RTs increased and (b) during easy search, there was no correlation between subjects’ overall RTs and the validity effect.

Because of a technical error, for one subject, each block included 108 instead of 80 trials per block. We verified that the results remained exactly the same when the data from this subject were removed from analysis.

Note that this effect reached significance when the analysis was conducted on raw RTs as in Gaspelin et al.’s (2016) study rather than on log-transformed RTs, F(1, 22) = 5.5, p = .03, *η^2^_p_* = .20.

This account seems to imply that actual shifts of attention occur only in the target display and not in the cue display, since the similarity of the distractors to the target contributes to priority allocation prior to attentional shifting. However, one may instead suggest that the temporal window across which attentional priority is accumulated before the competition is resolved, spans over a longer interval than the typical 150 ms cue-target SOA used in spatial cuing tasks, and that priority weights are updated online as events unfold. One may alternatively suggest that observers deploy their attention only as soon as a relevant event is detected (e.g., in Experiment 2: a letter, or the color red). Further research is required to address this issue.

## References

[1] Ansorge, U., Horstmann, G., & Scharlau, I. (2011). Top-down contingent feature-specific orienting with and without awareness of the visual input. Advances in Cognitive Psychology, 7, 108 DOI: 10.2478/v10053-008-0087-z 22253673 PMC3260021

[2] Bacon, W. F., & Egeth, H. E. (1994). Overriding stimulus-driven attentional capture. Perception & psychophysics, 55(5), 485–496. DOI: 10.3758/BF03205306 8008550

[3] Carmel, T., & Lamy, D. (2014). The same-location cost is unrelated to attentional settings: an object-updating account. Journal of experimental psychology: human perception and performance, 40(4), 1465–1478. DOI: 10.1037/a0036383 24730745

[4] Desimone, R., & Duncan, J. (1995). Neural mechanisms of selective visual attention. Annual Review of Neuroscience, 18, 193–222. DOI: 10.1146/annurev.ne.18.030195.001205 7605061

[5] Eimer, M., & Kiss, M. (2008). Involuntary attentional capture is determined by task set: Evidence from event-related brain potentials. Journal of cognitive neuroscience, 20(8), 1423–1433. DOI: 10.1162/jocn.2008.20099 18303979 PMC2564114

[6] Eriksen, C. W., & Yeh, Y. Y. (1985). Allocation of attention in the visual field. Journal of Experimental Psychology: Human Perception and Performance, 11(5), 583–597. DOI: 10.1037/0096-1523.11.5.583 2932532

[7] Faul, F., Erdfelder, E., Buchner, A., & Lang, A. G. (2013). G* Power Version 3.1. 7 [computer software] Uiversität Kiel, Germany.

[8] Folk, C. L., & Remington, R. W. (2006). Top-down modulation of preattentive processing: Testing the recovery account of contingent capture. Visual Cognition, 14, 445–465. DOI: 10.1080/13506280500193545

[9] Folk, C. L., & Remington, R. W. (2015). Unexpected abrupt onsets can override a top-down set for color. Journal of experimental psychology: human perception and performance, 41(4), 1153–1165. DOI: 10.1037/xhp0000084 26030438

[10] Folk, C. L., Remington, R. W., & Johnston, J. C. (1992). Involuntary covert orienting is contingent on attentional control settings. Journal of Experimental Psychology Human Perception and Performance, 18, 1030–1044. DOI: 10.1037/0096-1523.18.4.1030 1431742

[11] Franconeri, S. L., & Simons, D. J. (2003). Moving and looming stimuli capture attention. Attention, Perception, & Psychophysics, 65(7), 999–1010. DOI: 10.3758/BF03194829 14674628

[12] Gaspelin, N., Ruthruff, E., & Lien, M. C. (2016). The problem of latent attentional capture: Easy visual search conceals capture by task-irrelevant abrupt onsets. Journal of Experimental Psychology: Human Perception and Performance, 42(8), 1104–1120. DOI: 10.1037/xhp0000214 26854530 PMC4977216

[13] Gibson, B. S., & Kelsey, E. M. (1998). Stimulus-driven attentional capture is contingent on attentional set for display-wide visual features. Journal of Experimental Psychology: Human Perception and Performance, 24, 699–706. DOI: 10.1037/0096-1523.24.3.699 9627409

[14] Jonides, J., & Yantis, S. (1988). Uniqueness of abrupt visual onset in capturing attention. Perception & Psychophysics, 43, 346–354. DOI: 10.3758/BF03208805 3362663

[15] Lamy, D., Leber, A., & Egeth, H. E. (2004). Effects of Task Relevance and Stimulus-Driven Salience in Feature-Search Mode. Journal of Experimental Psychology: Human Perception and Performance, 30(6), 1019–1031. DOI: 10.1037/0096-1523.30.6.1019 15584812

[16] Lamy, D., Leber, A. B., & Egeth, H. E. (2012). Selective Attention In: Healy, A. F., & Proctor, R. W. (eds.), Experimental Psychology. Volume 4 in I.B. Weiner (Editor-in-Chief), Handbook of Psychology. New York: Wiley.

[17] Neo, G., & Chua, F. K. (2006). Capturing focused attention. Perception & Psychophysics, 68, 1286–1296. DOI: 10.3758/BF03193728 17378415

[18] Noesen, B., Lien, M. C., & Ruthruff, E. (2014). An electrophysiological study of attention capture by salience: Does rarity enable capture? Journal of Cognitive Psychology, 26(3), 346–371. DOI: 10.1080/20445911.2014.892112

[19] Prinzmetal, W., Ha, R., & Khani, A. (2010). The mechanisms of involuntary attention. Journal of Experimental Psychology: Human Perception and Performance, 36(2), 255–267. DOI: 10.1037/a0017600 20364917

[20] Rauschenberger, R. (2003). Attentional capture by auto- and allo-cues. Psychonomic Bulletin & Review, 10, 814–842. DOI: 10.3758/BF03196545 15000532

[21] Schreij, D., Owens, C., & Theeuwes, J. (2008). Abrupt onsets capture attention independent of top-down control settings. Perception & Psychophysics, 70(2), 208–218. DOI: 10.3758/PP.70.2.208 18372744

[22] Schreij, D., Theeuwes, J., & Olivers, C. N. (2010). Abrupt onsets capture attention independent of top-down control settings II: Additivity is no evidence for filtering. Attention, Perception, & Psychophysics, 72, 672–682. DOI: 10.3758/APP.72.3.672 20348574

[23] Theeuwes, J. (2010). Top-down and bottom-up control of visual selection. Acta psychologica, 135(2), 77–99. DOI: 10.1016/j.actpsy.2010.02.006 20507828

[24] Theeuwes, J., Atchley, P., & Kramer, A. F. (2000). On the time course of top-down and bottom-up control of visual attention. Control of cognitive processes: Attention and performance XVIII, 105–124.

[25] Wu, S. C., Remington, R. W., & Folk, C. L. (2014). Onsets do not override top-down goals, but they are responded to more quickly. Attention, Perception, & Psychophysics, 76, 649–654. DOI: 10.3758/s13414-014-0637-z 24596080

[26] Zivony, A., & Lamy, D. (in press). Contingent attentional engagement: stimulus- and goal-driven capture have qualitatively different consequences. Psychological Science. 10.1177/0956797618799302 30285577

