# Correction: Testing the Attentional Dwelling Hypothesis of Attentional Capture

**DOI:** 10.5334/joc.60

**Published:** 2019-03-26

**Authors:** Dominique Lamy, Maia Darnell, Adva Levi, Carmel Bublil

**Affiliations:** 1 Tel Aviv University, IL

**Keywords:** Attention, Visual search, Visual perception

## Abstract

This article details a correction to the article: Lamy, D., Darnell, M., Levi, A., & Bublil, C. (2018). Testing the Attentional Dwelling Hypothesis of Attentional Capture. *Journal of Cognition, 1*(1), 43. DOI: http://doi.org/10.5334/joc.48

## Correction

After publication the authors realized that in the study by Lamy, Darnell, Levi and Bublil (2018) there were two errors. (1) In Experiment 1, in Figure 3 and Table 2, the compatibility effects on the accuracy measure were mistakenly copy-pasted from the validity effects and therefore did not match the average accuracy rates reported in the text and there was a typo on p.8. The corrected Figure 3, Table 2 and text are provided below. (2) In Experiment 2, there was an error in the exploratory analyses because the computation of the compatibility effect was wrong. This error did not alter the conclusions of the paper but had an impact on the following parts of the article. The corrected text, table and figures are provided below.^1^

**Figure 3:**
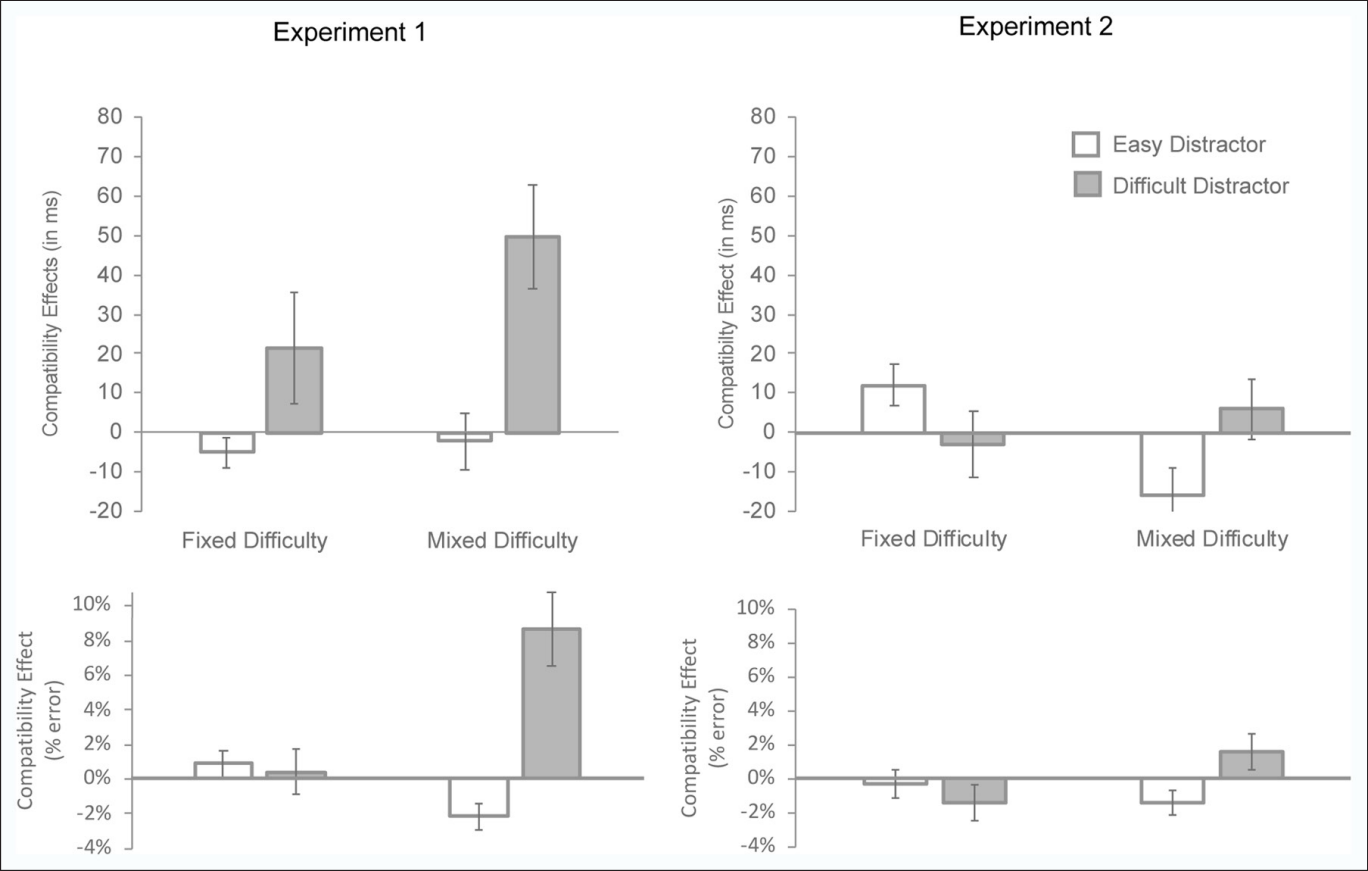
Mean compatibility effect (incompatible minus compatible) by condition of search type (fixed difficulty vs. mixed difficulty) and distractor difficulty (easy vs. difficult). *Upper panel*: Mean effect of reaction times (in milliseconds). *Lower panel*: Mean effect on error rates (in percentage).

**Table 2:** Mean RTs (in milliseconds) and accuracy rates (in percentage) on invalid-cue trials in Experiment 1 by conditions of cue-target compatibility, cued-distractor difficulty and search type. The numbers between square brackets represent the standard errors.

	All-easy		All-difficult		Mixed-easy		Mixed-difficult	

*Reaction times*								
Compatible	640	[23]	904	[31]	759	[23]	753	[23]
Incompatible	635	[21]	925	[30]	757	[25]	803	[24]
*Accuracy*								
Compatible	96.3%	[1.0%]	93.7%	[1.5%]	91.9%	[1.4%]	96.4%	[0.8%]
Incompatible	95.4%	[1.0%]	93.3%	[1.7%]	94.0%	[0.9%]	87.8%	[2.0%]

*Page 8, Figure 3*.

*Page 8, Table 2*.

*Page 8*, When distractor difficulty was mixed, the interaction was significant, F(1, 23) = 10.4, p = .0038, *η^2^_p_* = .31, indicating that the compatibility effect was larger when the cued distractor was difficult, 50 ms, F(1, 23) = 13.5, p = .0013, *η^2^_p_* = .37 than when it was easy, –2 ms, F < 1.

*Page 11, results in the section entitled Exploratory analyses: compatibility effects*.

*Reaction times.* The main effect of cued distractor difficulty was significant, F(1, 22) = 71.8, p < .0001, *η^2^_p_* = .77, whereas the main effects of compatibility and search type were not, F < 1 and F(1, 22) = 3.11, p = .09, *η^2^_p_* = .12, respectively. The three-way interaction, between cued distractor difficulty, search type and compatibility was significant, F(1, 22) = 7.59, p = .01, *η^2^_p_* = .26. Follow-up comparisons revealed no compatibility effect when the cued distractor was difficult, in either the fixed- or the mixed-difficulty search, –3 ms and 7 ms, respectively, both Fs < 1. When the cued distractor was easy, the compatibility effect was positive in the fixed-difficulty search, 11 ms, F(1, 22) = 6.53, p = .02, *η^2^_p_* = .23 and negative in the mixed-difficulty search, –16 ms, F(1, 22) = 5.37, p = .03, *η^2^_p_* = .20.

*Accuracy.* Only the three-way interaction approached significance, F(1, 22) = 3.48, p =.08, *η^2^_p_* = .14. None of the paired comparisons reached significance.

*Page 11, Table 4*.

**Table 4:** Mean RTs (in milliseconds) and accuracy rates (in percentage) on invalid-cue trials in Experiment 2 by conditions of cue-target compatibility, cued-distractor difficulty and search type. Standard errors are reported in brackets.

	All-easy		All-difficult		Mixed-easy		Mixed-difficult	

*Reaction times*								
Compatible	640	[23]	904	[31]	759	[23]	753	[23]
Incompatible	635	[21]	925	[30]	757	[25]	803	[24]
*Accuracy*								
Compatible	96.3%	[1.0%]	93.7%	[1.5%]	91.9%	[1.4%]	96.4%	[0.8%]
Incompatible	95.4%	[1.0%]	93.3%	[1.7%]	94.0%	[0.9%]	87.8%	[2.0%]

p.12, discussion of Experiment 2, second paragraph summarizing the compatibility effects.

Compatibility effects (i.e., better performance when the responses associated with the cued distractor and target were compatible than when they were incompatible) were very weak and inconsistent in this experiment. There was no compatibility effect when the difficult distractor was cued. Inconsistent effects emerged in the easy distractor condition. Such absence of compatibility effects in this experiment relative to Experiment 1 is likely to result from the much weaker location effect (i.e., better performance when the target appeared at the cued location than at a non-cued location) associated with the abrupt onset cue. While a location effect was observed in all conditions in Experiment 1, it was found only in the all-difficult condition in Experiment 2, and was much smaller than in the all-difficult condition of Experiment1 (26 ms vs. 121 ms, respectively).

p.13, General Discussion, penultimate paragraph of the section entitled: “A re-evaluation of the attentional dwelling hypothesis”.

Finally, the dwelling hypothesis does not explain the full pattern of results that arose from exploratory analyses of the compatibility effect, in particular, why a compatibility effect was associated with the difficult distractor on trials in which an easy distractor was cued in the mixed-difficulty search condition of Experiment 1.

*p.14, General Discussion, 2^nd^ paragraph of the section entitled: “Contradiction with the findings reported by Zivony and Lamy (in press)”*.

The present findings challenge these conclusions because we found instances of compatibility effects following irrelevant-color onset cues, namely, in the mixed-difficulty search condition of Experiment 1.

## Data Accessibility Statement

The corrected data file can be found at https://doi.org/10.6084/m9.figshare.7770941.v2

## Footnotes

The mean compatibility effects on RTs and accuracy presented in the graphs and tables below were calculated per condition of cued distractor difficulty and search type. The mean compatibility effects across each of these factors reported in the text original manuscript (e.g., on page 7) do not correspond to the arithmetic average of the relevant cells because they factor in the imbalance in the number of trials in the different conditions. For instance, in the mixed-difficulty condition, there were two easy distractors and only one difficult distractor. Thus, the compatibility effect from easy distractors had more weight than the compatibility effect from difficult distractors in the compatibility effect reported for the fixed- vs. mixed search (across conditions of cued distractor difficulty).

## References

[1] Lamy, D., Darnell, M., Levi, A., & Bublil, C. (2018). Testing the Attentional Dwelling Hypothesis of Attentional Capture. Journal of Cognition, 1(1), 43 DOI: 10.5334/joc.48 PMC6634340 31517216

